# Surgical outcomes after reoperation of intra-articular proximal ulna fractures

**DOI:** 10.1016/j.jseint.2024.12.017

**Published:** 2025-01-23

**Authors:** Nadia Azib, Huub H. de Klerk, Michel P.J. van den Bekerom, Abhiram R. Bhashyam

**Affiliations:** a Hand and Arm Research Collaborative, Massachusetts General Hospital, Harvard Medical School, Boston, MA, USA; b Department of Orthopaedic Surgery, OLVG, Amsterdam, the Netherlands; c Amsterdam Shoulder and Elbow Center of Expertise (ASECE), OLVG, Amsterdam, the Netherlands; d Department of Orthopaedic Surgery, University Medical Centre Groningen (UMCG) and Groningen University, Groningen, the Netherlands; e Faculty of Behavioural and Movement Sciences, Vrije Universiteit Amsterdam, Amsterdam, the Netherlands; f Harvard Medical School, Boston, MA, USA

**Keywords:** Proximal ulna, Intra-articular, Reoperation, Range of motion, Complications, Shared decision-making

## Abstract

**Background:**

Literature on outcomes after reoperation for intra-articular proximal ulna fractures is lacking, even though reoperation rates for these fractures are reportedly one of the highest of any anatomic site, ranging from 22%-89%. This study aims to evaluate range of motion (ROM) and complication rates after reoperation for these fractures.

**Methods:**

In this retrospective single institution cohort study, we identified 134 patients with intra-articular, comminuted fractures of the proximal ulna initially treated with open reduction internal fixation between January 2015 and March 2022. Of this cohort, 34 of the 134 patients (25%) underwent reoperation. A Wilcoxon signed-rank test was conducted to assess differences between preoperative and postoperative ROM.

**Results:**

Symptomatic hardware was the most common indication for reoperation (28/34 [82%]), followed by ulnar neuropathy (4/34 [12%]). ROM remained similar before and after reoperation for patients who underwent reoperation for indications other than stiffness. Patients that were reoperated for stiffness showed a 9° (*P* = .03) improvement in extension and 26° (*P* = .02) improvement for flexion. Twelve patients experienced complications, of which persistent implant irritation (3/12 [25%]) and tendinopathy (3/12 [25%]) were the most common.

**Conclusion:**

In our study cohort, 25% of patients underwent reoperation—most often due to symptomatic hardware. While ROM is typically preserved after reoperation and improved when the indication for reoperation is elbow stiffness, a significant proportion of patients (35%) experience subsequent complications. Counseling patients about reoperation outcomes is essential to manage patient expectations and can help them make informed decisions. This approach supports informed decision-making and optimizes patient care.

Comminuted proximal ulna fractures are challenging to treat.^1^^,^^3^^,^^17^ Previous studies have noted complication rates between 46%-47%, of which 36%-56% require reoperation.^11^^,^^13^^,^^27^ The most common reason for reoperation after open reduction and internal fixation (ORIF) of proximal ulna fractures is symptomatic hardware.^5^^,^^12^^,^^13^ Reported rates range from 22%-89% for plate fixation and up to 50% for tension band wiring,^12^^,^^18^^,^^27^^,^^36^ making it one of the highest implant removal rates across all anatomic sites.^13^ Approximately 1.4%-56% of reoperations for comminuted proximal ulna fractures are specifically undertaken to alleviate elbow contractures and improve range of motion (ROM).^20^^,^^28^^,^^31^^,^^32^^,^^33^ Elbow stiffness is a major problem which can lead to symptoms of depression and anxiety^21^ and perceived disability in daily activities.^20^ A recent study demonstrated that decreases in the ROM flexion-extension arc strongly associated with decreased patient satisfaction.^6^

Although reoperations after ORIF of proximal ulna fractures are common, there is limited literature about how patients function after reoperation. This knowledge gap can make it difficult to accurately counsel patients about the benefits and risks of reoperation, especially when reoperation is frequently performed for more elective indications, such as symptomatic hardware.^19^ Therefore, our study aimed to 1) evaluate ROM after reoperation, 2) assess whether ROM, specific fracture patterns, and extent of comminution are associated, and 3) evaluate complication rates after reoperation.

## Materials and methods

This is a retrospective cohort study evaluating patients with intra-articular, comminuted fractures of the proximal ulna. This study was approved by our institution’s institutional review board and was done according to the Strengthening the Reporting of Observational Studies in Epidemiology guidelines^14^ (Supplementary Table S1).

### Patient selection

**Figure 1:**
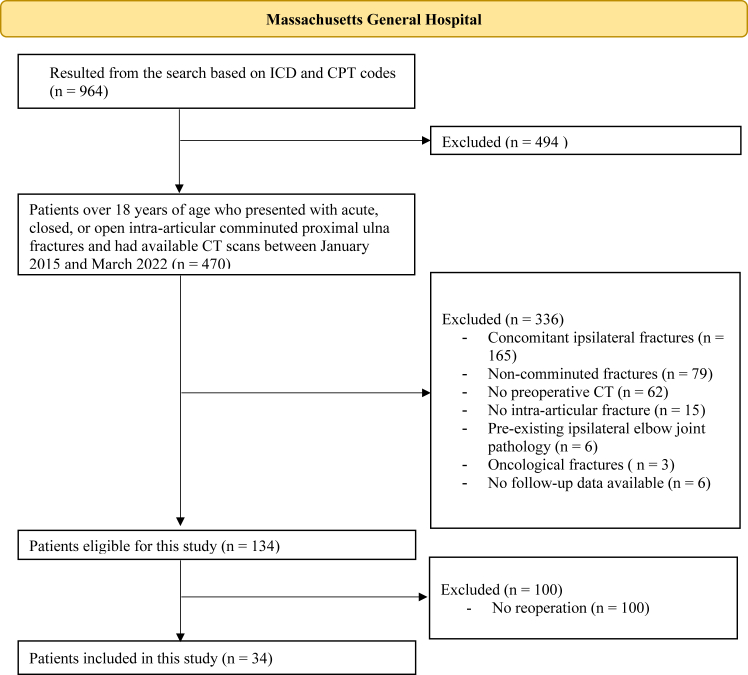
Overview of patient inclusion and exclusion criteria. *ICD*, International Classification of Diseases; *CPT*, Current Procedural Terminology; *CT*, computed tomography.

Our electronic health database was initially searched with International Classification of Diseases (ICD-9/10) codes and Current Procedural Terminology codes: (1) for proximal ulna fractures, (2) with a computed tomography (CT) scan of the elbow, and (3) who underwent surgical treatment between January 2015 and March 2022. Subsequent International Classification of Diseases and Current Procedural Terminology codes can be found in Supplementary Table S2. From this initial screen of 964 patients, we then identified 470 adult patients (>18 years of age) who had presented with acute, closed, or open intra-articular, comminuted proximal ulna fractures. Patients with concomitant ipsilateral fractures (n = 165), noncomminuted fractures (n = 79), no preoperative CT (n = 62), no intra-articular fracture (n = 15), pre-existing ipsilateral elbow joint pathology (n = 6), and oncological fractures (n = 3) were excluded. Six more patients were excluded since their primary surgery was conducted at an outside institution without follow-up data available to us (Fig. 1). Excluding patients without CT scans ensured accurate fracture characterization. Additionally, the exclusion of ipsilateral injuries minimized confounding factors that could influence complication rates and outcomes.

### Baseline patient, injury, and treatment characteristics

**Figure 2:**
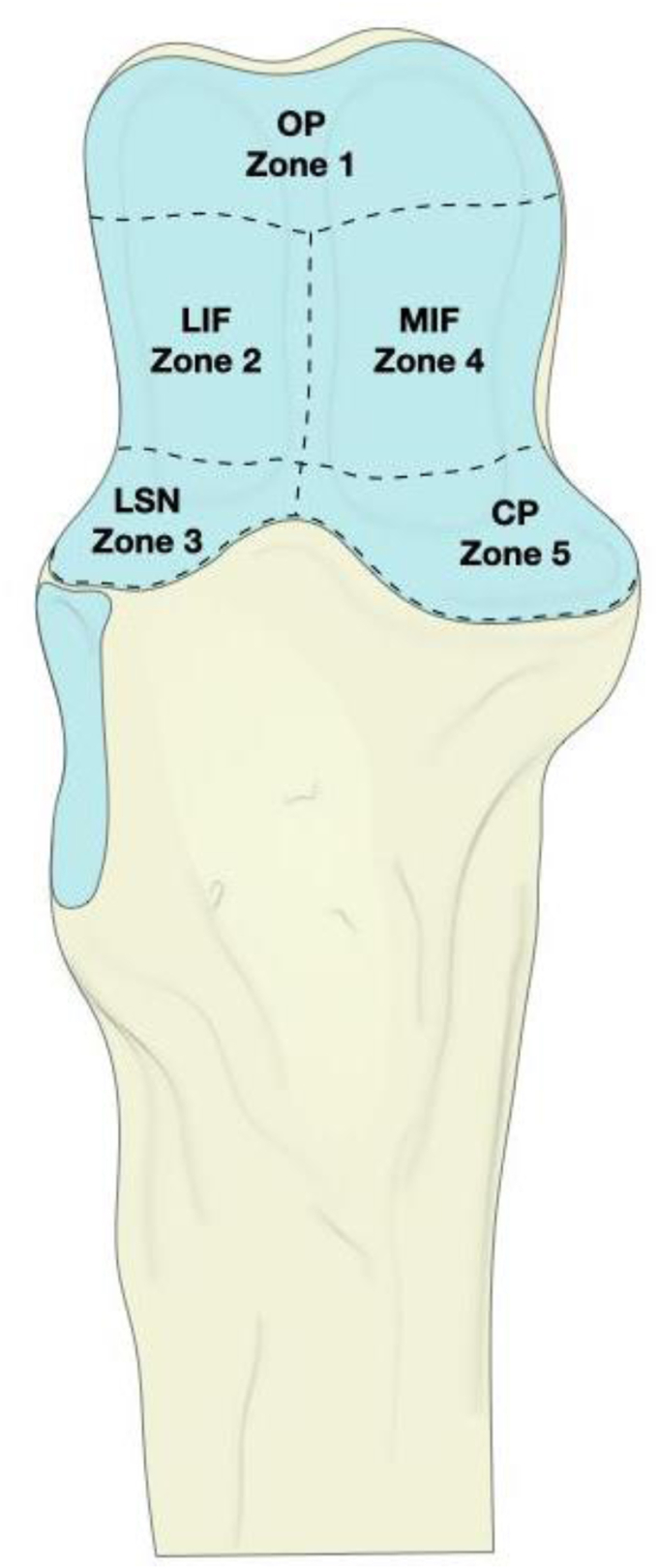
Three-dimensional image of the articular surface of the proximal ulna divided into the 5 described zones. *OP*, olecranon process; *LIF*, lateral intermediate facet; *LSN*, lesser sigmoid notch; *MIF*, medial intermediate facet; *CP*, coronoid process.

Baseline variables collected from the electronic medical record included age, sex, body mass index, injury characteristics (number of involved fracture zones, Mayo fracture type), and treatment characteristics (indication for reoperation, primary surgery duration, follow-up time from initial surgery). To assess the number of involved fracture zones, we modified the classification used by Chapleau et al.^9^ Fractures of the sublime tubercle and anteromedial facet were combined into one zone (zone 5), while posterior and intermediate fragments were combined and then divided into medial and lateral fractures (zones 2 and 4). The articular space of the proximal ulna was divided into 5 zones based on soft tissue insertions—the olecranon process (zone 1), the lateral intermediate facet (zone 2), the lesser sigmoid notch (zone 3), the medial intermediate facet (zone 4), and the coronoid fragment (zone 5) (Fig. 2). This zonal division allows for a more fragment-specific approach of categorizing fractures, aligning fracture locations with the anatomical regions critical for joint stability and movement. The Mayo classification was used to categorize patients into 3 groups: Mayo type 1 (nondisplaced), Mayo type 2 (displaced and stable), and Mayo type 3 (displaced and unstable).^35^ Since all fractures were comminuted, all Mayo types were classified as the B-subtype. All cases were independently classified by two of the authors (N.A., H.K.), and disagreement was resolved by discussion with the senior author (A.B.).

### Outcomes

Outcomes included final ROM, complications after the reoperation, and Visual Analog Scale (VAS) score for pain.

ROM was collected before and after reoperation from the electronic medical record when patients underwent reoperation, and the proportion of patients who achieved sufficient ROM for activities of daily living (ADL) was calculated. ROM sufficient for ADL was considered to be 130° for flexion, 30° for extension, and 50° for both supination and pronation, according to Morrey et al.^24^ A subgroup of patients had elbow stiffness following primary surgery. Stiffness was defined as flexion contracture >30°, <120° of flexion, and <50° of supination and pronation.^26^^,^^33^

Complications were categorized as minor and major by the senior author based on the severity of the complications, aligning with previous literature.^23^^,^^38^ Classifying complications as minor or major helps the physician to clearly communicate the potential risks associated with a reoperation during shared decision-making. This categorization allows patients to understand the likelihood of complications, as well as their potential severity. Major complications were defined as events that significantly impact quality of life and that may require more intensive treatment or surgical intervention. All other complications were considered minor. The VAS was collected from patient notes following reoperation and was used to report pain on a range from 0-10, where 0 represents “no pain” and 10 indicates “the worst pain imaginable.” Regarding operation indication, symptomatic hardware was defined as an implant that caused considerable irritation or might warrant removal on assessment made by the treating physician.

### Statistical analysis

All statistical analyses were conducted using Stata Statistical Software (Release 18; StataCorp LLC, College Station, TX, USA).^34^ Descriptive univariate analysis was used for baseline characteristics, including mean, standard deviation, and range for continuous variables (age, pre- and postoperative ROMs, primary surgery duration, and follow-up from primary surgery on) and frequency distributions tables for complications. All outcomes were collected following primary and secondary surgery. Since patients could present with fractures in multiple anatomical zones, each injury was counted only once to avoid double-counting and to accurately represent fracture distribution within this patient population. Complications and reoperations were recorded as binary variables.

Chi-squared tests were used to assess the association between fracture classifications and reoperation rate. McNemar’s chi-squared test was also used to look at the paired proportion of ADL sufficiency in the reoperation group. A Wilcoxon signed-rank test was used to assess if there was a difference between preoperative and postoperative ROM, and delta (Δ) was used to indicate the difference in ROM. Nonparametric statistical tests were used due to the non-normal distribution of the data as identified by histogram analysis. Complete case analysis was used as imputation methods would not be robust given the heterogeneity of injuries. The significance level (alpha) was set at 0.05.

## Results

### Patient characteristics

A total of 134 patients were included for analysis, of which 34 (25%) underwent reoperation. No difference was seen in demographics between the patients who underwent a reoperation and those who did not undergo a reoperation (Table I). The number of fractured zones was not associated with the likelihood of reoperation (*P* = .79), and neither was the Mayo classification (*P* = .56).

**Table I:** Baseline characteristics.

Characteristics	No reoperation *(n = 100)*	Underwent reoperation *(n = 34)*	Total *(n = 134)*	*P* value
Gender, female: *n (%)*	59 (59)	22 (65)	81 (60)	.56
Age, y: *mean ± SD*	54 ± 20	54 ± 17	54 ± 19	.98
Side of injury, right: *n (%)*	52 (52)	14 (41)	66 (49)	.28
Body mass index, *mean ± SD*	26 ± 6	25 ± 5	26 ± 5	.26
Primary surgery duration, minutes: *mean ± SD*	133 ± 69∗	136 ± 77	134 ± 71	.83
Follow-up from primary surgery on, mo: *mean ± SD*	11 ± 13	16 ± 18	12 ± 15	.12
Dementia: *n (%)*	5 (5)	1 (3)	6 (5)	.62
Diabetes: *n (%)*	8 (8)	4 (12)	12 (9)	.51
Osteoporosis: *n (%)*	32 (32)	14 (41)	46 (34)	.33
Radial fracture: *n (%)*	38 (38)	14 (41)	52 (39)	.74
Number of fractured zones *n (%)*				
1	5 (5)	1 (3)	6 (4)	.79
2	10 (10)	2 (6)	12 (9)	
3	44 (44)	19 (56)	63 (47)	
4	15 (15)	4 (12)	19 (14)	
5	26 (26)	8 (24)	34 (25)	
Mayo classification†*n (%)*				
1B	15 (15)	5 (15)	20 (15)	.56
2B	53 (54)	21 (64)	74 (55)	
3B	30 (31)	7 (21)	37 (28)	

*n*, number of patients; *SD*, standard deviation.

∗ Eighty-seven patients, 13 missing values due to either nonoperative treatment or no documentation.

† One hundred thirty-one patients, 3 missing values as no Mayo type could be appointed due to lack of olecranon fracture.

### Indications for reoperation

Symptomatic hardware was the most common indication for reoperation (28/34 [82%]), followed by ulnar neuropathy (4/34 [12%]). Primary reasons for implant removal were secondary to implant irritation (24/28 [86%]), infection (3/28 [10%]), and fragment displacement (1/28 [4%]). Prior to reoperation, 2 patients (2/34 [6%]) experienced nonunion of the proximal ulna, one patient (1/34 [3%]) had a loss of reduction, and one patient (1/34 [3%]) experienced recurrent dislocation. Twenty-three percent of the patients (31/134) experienced a restricted ROM which could be classified as functional elbow stiffness after initial surgery, of which nine patients (27%) underwent a reoperation.

### Range of motion

In the subset of patients who underwent reoperation, postoperative ROM for extension improved minimally (Δ: 3°, *P* = .025). There was no observed difference between the preoperative and postoperative ROM for flexion (Δ: 8°, *P* = .18), supination (Δ: −1°, *P* = .32), or pronation (Δ: 1°, *P* = .68). The percentage of patients achieving the required ROM for ADL remained unchanged for flexion (68% vs. 73%; *P* = .66), extension (91% vs. 100%; *P* = .16) supination (100% vs. 94%; *P* = .32), and pronation (94% vs. 89%; *P* = 1.00) (Table II).

**Table II:** Mean range of motion after reoperation in total cohort.

Patients∗	Preoperative mean ROM (degrees ± SD)	Postoperative mean ROM (degrees ± SD)	*P* value	Preoperative sufficient for ADL†*(n [%])*	Postoperative sufficient for ADL†*(n [%])*	*P* value
Flexion, n = 22	124 ± 26	132 ± 10	.18	15 (68)	16 (73)	.66
Extension, n = 22	12 ± 13	9 ± 9	**.03**	20 (91)	22 (100)	.16
Supination, n = 17	83 ± 12	82 ± 13	.32	17 (100)	16 (94)	.32
Pronation, n = 18	82 ± 17	83 ± 13	.68	17 (94)	16 (89)	1.00

*n*, number of patients; *ROM*, range of motion; *SD*, standard deviation; *ADL*, activities of daily living.

*P* values in bold are statistically significant.

∗ Patients were only included in the analysis when both preoperative ROM and postoperative ROM were reported.

† Sufficient for ADL, is considered 130 degrees for flexion, 30 degrees for extension, and 50 degrees for both supination and pronation.^26^

Among the patients who underwent reoperation, nine had pre-existing elbow stiffness in addition to their primary indication for the procedure, such as symptomatic hardware or ulnar neuropathy. In this subset of patients, reoperation increased average flexion by 26° (*P* = .02) and improved average extension by 9° (*P* = .03). ROM for supination (Δ: −2°, *P* = .32) and pronation (Δ: −2°, *P* = .84) were unchanged after reoperation. In this cohort, reoperation resulted in improvements in ADL sufficiency in two patients (22%) for both flexion and extension, while one patient (11%) demonstrated improvement in pronation following reoperation. Patients who underwent reoperation for other reasons maintained their preoperative ROM postoperatively (Table III).

**Table III:** Outcomes for reoperations for patients with and without elbow stiffness based on latest reported preoperative ROM.

	Preoperative mean ROM (degrees ± SD)	Postoperative mean ROM (degrees ± SD)	*P* value
Patients without elbow stiffness *(n [%])*∗			
Flexion, n = 15	136 ± 7	135 ± 8	.42
Extension, n = 15	7 ± 6	6 ± 5	.29
Supination, n = 14	85 ± 9	83 ± 13	.08
Pronation, n = 14	86 ± 7	84 ± 12	.54
Patients with elbow stiffness *(n [%])*∗			
Flexion, n = 7	99 ± 34	125 ± 10	**.02**
Extension, n = 7	24 ± 16	15 ± 12	**.03**
Supination, n = 3	70 ± 17	73 ± 15	.32
Pronation, n = 4	68 ± 32	80 ± 14	.84

*n*, number of patients; *ROM*, range of motion; *SD*, standard deviation.

*P* values in bold are statistically significant.

∗ Patients were only included in the analysis when both preoperative ROM and postoperative ROM were reported.

### Fracture patterns

We found that an increase in the number of fracture fragments was associated with a decrease in both flexion (Δ: 12°, *P* = .01) and supination (Δ: 17°, *P* = .01), but not extension (Δ: 6°, *P* = .11) or pronation (Δ: 8°, *P* = .27) (Table IV). Fractures associated with elbow instability (Mayo type 3 fractures) were associated with a decrease in flexion (Δ: 10°, *P* = .05), supination (Δ: 20°, *P* = .01), and pronation (Δ: 14°, *P* = .03), but not with extension (Δ: 1°, *P* = .48) (Table IV).

**Table IV:** Relation of the number of fracture zones and Mayo type fracture on range of motion after reoperation.

		Flexion	Extension	Supination	Pronation
		Mean (n) *[N = 26]*	Mean (n) *[N = 27]*	Mean (n) *[N = 22]*	Mean (n) *[N = 22]*
Number of fractured zones *(n)*∗	1 + 2 + 3	133 (16)	10 (17)	85 (14)	86 (14)
	4 + 5	121 (10)	16 (10)	68 (8)	78 (8)
*P* value		**.01**	.11	**.01**	.27

		**Flexion**	**Extension**	**Supination**	**Pronation**
		Mean (n) *[N = 25]*	Mean (n) *[N = 26]*	Mean (n) *[N = 21]*	Mean (n) *[N = 21]*
Mayo classification *(n)*∗	1B + 2B	131 (18)	13 (19)	85 (15)	88 (15)
	3B	121 (7)	12 (7)	65 (6)	74 (6)
*P* value		**.0498**	.48	**.01**	**.03**

*n*, number of patients; *ROM*, range of motion.

*P* values in bold are statistically significant.

∗ Patients were only included in the analysis when postoperative ROM was reported.

### Complications

Twelve of the 34 reoperated patients (35%) experienced a total of 20 complications (Table V). The most common minor complications observed were symptomatic implants and tendinopathy, occurring in 3 patients each (3/34 [9%]). The most common major complications were deep infection and wound complications, each affecting 2 patients (2/34 [6%]). The VAS for pain was reported in 27 patients, with a mean postoperative VAS of 2.8 ± 2.5.

**Table V:** Complication rates, divided into major and minor complications, after reoperations.

Complications			
Minor complications, n (%)∗		Major complications, n (%)∗	
Persistent implant irritation	3 (9)	Deep infection	2 (6)
Tendinopathy	3 (9)	Wound complications	2 (6)
Edema	2 (5)	Persistent nonunion	1 (3)
Intraoperative implant breakage	1 (3)	Bilateral pulmonary emboli	1 (3)
Olecranon bursitis	1 (3)	Persistent ulnar nerve neuropathy	1 (3)
Persistent paresthesia	1 (3)	Ulnohumeral arthritis	1 (3)
New onset, transient, ulnar nerve neuropathy	1 (3)		
**Total**	**12 (35)**	**Total**	**8 (24)**

*n*, number of patients.

∗ Percentages displayed are calculated by dividing the number of complications by the total number of reoperations (n = 34).

## Discussion

In this study, we found that reoperation after ORIF of comminuted intra-articular proximal ulna fractures maintains or improves average ROM and increases the proportion of patients capable of performing ADLs, especially in settings of clinically significant elbow stiffness. Patients with more significant intra-articular comminution or elbow instability often presented with greater restrictions in flexion and supination. After reoperation, the entire study cohort showed only minimal improvements in extension ROM, with no significant changes in flexion, supination, or pronation. However, when focusing on patients who had preoperative elbow stiffness, an improvement in both flexion and extension was seen; these patients did not show any changes in supination or pronation. These findings indicate that ROM is not compromised by reoperation. In fact, in cases of initial elbow stiffness, ROM in the flexion-extension arc can actually improve following reoperation.

### Strengths and limitations

Key strengths of this study include its carefully selected, uniform population, resulting from single-site sampling and stringent inclusion criteria that isolate a specific subset of proximal ulna fractures. Additionally, the study draws on high-quality references, featuring numerous respected clinical trials and review articles. This study has several limitations—many are inherent to the retrospective study design. First, our study cohort for reoperation was 34 patients identified over a 7-year period, but this is still a relatively small sample size for robust statistical analysis due to the stringent inclusion criteria. The initial query was intentionally broad to ensure no potential patients were overlooked. It was subsequently refined to focus on the study group, thereby avoiding the comparisons involving patients and fractures in different contexts. We believe that this methodological approach enhanced both the internal and external validity of the study. While we plan to investigate patients with ipsilateral injuries in future research, such injuries are not pertinent to the specific scope of this study. Additionally, we excluded patients with concomitant ipsilateral fractures and those without preoperative CT imaging to establish a homogeneous cohort focused specifically on isolated intra-articular comminuted proximal ulna fractures, ensuring CT imaging was available for accurate evaluation of coronoid fracture types.^37^ While this exclusion minimized confounding factors, it also limited the generalizability of our findings to a broader population. Future studies could explore the impact of ipsilateral injuries and missing CT scans on complication and reoperation rates to better understand how these factors might influence outcomes in this patient group. Nevertheless, existing literature on this population is very limited, so our results can still be used to identify trends, patterns, or potential associations that can guide further research, especially because reoperation is so common in this population. Secondly, our database did not consistently have patient-reported outcome measures (PROMs) recorded precluding analysis. PROMs are valuable for understanding the intervention's impact from the patient's perspective and facilitating shared decision-making. Reporting PROMs for this patient population is an area for future research as they may differ from those associated with more common elbow fracture patterns. Finally, due to the retrospective design of this study, we cannot ensure the consistent application of a standardized method for measuring the ROM. Nonetheless, it is standard practice in our institution to utilize a goniometer for assessing ROM.

### Indications for reoperation

Reported rates for implant removal range from 22%-89% for plate fixation and up to 50% for tension band wiring.^12^^,^^18^^,^^27^^,^^36^ In our study, symptomatic implants were the most common indication for reoperation, accounting for 80% of cases. Primary reasons for implant removal were secondary to implant irritation (86%), infection (10%), and fragment displacement (4%). These reasons, although elevated in our population, mostly coincide with those reported by other studies. Anantavorasakul et al^2^ reported rates of 64% and 8% for implant irritation and stiffness, respectively, while Bachoura et al^4^ reported reoperations for elbow stiffness at 5%, with infection and symptomatic implants each at 33%. Implant removal is often elective and based on shared decision-making between surgeons and patients. For this reason, having accurate information about the expected outcome is critical. For example, Edwards et al^13^ found that 73% of patients would undergo a reoperation for hardware removal if guaranteed a safe procedure. Furthermore, in a recent study by Hambrecht et al,^15^ only a minority of patients had improvement in ROM (28% in olecranon fractures and 40% in radial fractures), but pre- and postoperative ROM were not provided for comparison. Only 50% of patients with olecranon fractures, and 49% of patients with radius fractures reported satisfaction with their outcomes.^15^ This underscores the significance of knowing the expected postoperative ROM and complication rate for effective patient counseling.

### Range of motion and stiffness

For the total cohort of patients undergoing reoperation, the mean postoperative extension improved by 3°, which, despite achieving statistical significance (*P* = .025), may not represent a clinically meaningful change. Given the small cohort, this improvement might be negligible in practical terms, as minor ROM differences like this are unlikely to impact functional outcomes meaningfully. However, the goniometer used to measure ROM has a measurement error of approximately 10°, which could influence the clinical interpretation of these small changes.^8^ Future studies with larger cohorts would help clarify the clinical relevance of small ROM changes postoperatively.

Elbow stiffness has a significant impact on the ability to complete ADL and overall patient satisfaction.^25^^,^^32^ However, not all patients with stiffness defined by ROM measurements are symptomatic, as symptoms may depend on the required ROM for patient-specific work activities, sports, and hobbies.^32^ In our study, reoperation for stiffness after ORIF of comminuted intra-articular proximal ulna fractures led to a 26° increase in flexion and enhanced extension by 9° on average; this is on the lower end of previous estimates of improvement of ROM after stiffness operations where the arc of motion improved by 40°-71°.^25^^,^^28^^,^^31^^,^^32^ To some degree, this may be because we only included comminuted intra-articular fractures in this study, and we found that an increase in the number of intra-articular fracture fragments was associated with a decrease in flexion-extension arc that likely affected articular congruity even after an elbow stiffness operation. Similarly, fractures associated with elbow instability also had a decrease in flexion-extension arc and rotation.

### Observed complication rate

Literature on outcomes after reoperation is scarce for intra-articular, comminuted fractures of the proximal ulna. De Giacomo et al^11^ reported a complication rate of 22% for 32 patients following reoperation in displaced olecranon fractures, primarily attributed to deep infections and wound dehiscence. We believe that our complication rate diverges from this due to our exclusive focus on comminuted, intra-articular fractures, known for their high risk of complications.^22^ Conversely, Rotini et al^29^ reported a complication rate of 33% for 12 patients after reoperations for proximal ulna nonunion, which included symptomatic hardware, transient ulnar nerve symptoms, and plate breakage. Sanderson et al^30^ also reported a higher complication rate of 42% in 24 patients with forearm fractures who underwent plate removal, in which infection and permanent nerve injury were the most dominant. Our observed complication rate of 35% appears to align with the above findings. Awareness of this complication rate is crucial for preparing patients for potential outcomes and ensuring vigilant postoperative monitoring, which in turn supports informed decision-making and optimizes patient care.

To contextualize our complication rate, we examined potential risk factors. However, due to the small sample size of our study, we lack the statistical power to draw definitive conclusions regarding the impact of age, body mass index, or sex on the likelihood of reoperation or complications. As such, we refer to existing literature for further insights. Previous studies suggest that a younger age,^10^^,^^27^ higher body mass index,^7^ and female sex^10^ is linked to higher complication and reoperation rates.

### Future research

A key area for future research is the detailed assessment of risk profiles for patients undergoing reoperations. Patients in our study cohort required reoperations due to complications from their initial procedures. This preselection bias suggests a predisposition to unfavorable outcomes.^16^ Therefore, a comprehensive evaluation of the factors contributing to these complications and the need for reoperation is essential. Understanding these risk profiles can lead to more accurate predictions and better management strategies, ultimately reducing the incidence of complications in this patient group. Future studies should focus on identifying specific predictors of complications to enhance preoperative assessments and tailor interventions accordingly.

## Conclusion

A lack of information on reoperation outcomes for comminuted proximal ulna fractures hampers informed decision-making. While ROM is typically preserved after reoperation and improved when the indication for reoperation is elbow stiffness, a significant proportion of patients (35%) experience subsequent complications. Symptomatic hardware was the most common indication for reoperation, underscoring the clinical challenges associated with hardware implantation. These data can help counsel patients who may require a reoperation after ORIF of a comminuted intra-articular proximal ulna fracture, especially in elective cases, such as symptomatic implants, elbow stiffness, or ulnar nerve irritation. Counseling patients about reoperation outcomes is essential to manage patient expectations and can help them make informed decisions. This approach supports informed decision-making and optimizes patient care.

## Disclaimers:

Funding: This work was in part supported by the Jesse B. Jupiter Research Fund of the Wyss Medical Foundation. Additionally, N.A. reports receipt of support by “Vreedefonds” (Amsterdam, the Netherlands) and “Hendrik Muller fonds” (the Hague, the Netherlands). H.K. reports receipt of support by De Stichting Prof. Michaël-van Vloten Fonds (the Hague, the Netherlands), Van Leersum Grant/KNAW Medical Sciences Fund from the Royal Netherlands Academy of Arts & Sciences (Amsterdam, the Netherlands), Marti-Keuning Eckhardt Foundation (Amsterdam, the Netherlands), Vreedefonds (Amsterdam, the Netherlands), the Stichting Anna Fonds | NOREF (Mijdrecht, the Netherlands), the Stichting het Scholten-Cordes Fonds (the Hague, the Netherlands), Fundatie van Renswoude (‘s-Gravenhage, the Netherlands), and the USC Scholarship Foundation (Utrecht, the Netherlands).

Conflicts of interest: The authors, their immediate families, and any research foundations with which they are affiliated have not received any financial payments or other benefits from any commercial entity related to the subject of this article.

## Supplementary Data

Supplemental 1 STROBE
