# Supplementary material for: Surgical outcomes after reoperation of intra-articular proximal ulna fractures
Source: JSES Int. 2025 Jan 23;9(3):885–92. doi: 10.1016/j.jseint.2024.12.017 (PMC12144971; doi:10.1016/j.jseint.2024.12.017)
Supplement: Supplemental 2 [file mmc2.docx]

**Supplemental 2:** International Classification of Diseases (ICD-9/10) codes and Current Procedural Terminology (CPT) codes, searched between January 2015 and March 2022.

| **Category** | **ICD/CPT code** |
| --- | --- |
| Proximal ulna fractures | ICD9: 81300-81304, 81308, 81310-81314, 81318, 81320, 81322-81323, 81330, 81332-81333, 81380, 81382-81383, 81390, 81392-81393 |
| CT scan of the elbow | CPT: 73200-73202, 73206, 73070, 73080, 73085, 73090 |
| Underwent surgical treatment | CPT: 24670, 24675, 24615, 24635, 24585-24587 |
